# P-613. Impact of Maternal Tdap Booster Vaccination During Pregnancy on Pertussis Incidence, Vaccine Effectiveness, Hospitalization, and Mortality in Newborns and Infants: A Systematic Literature Review

**DOI:** 10.1093/ofid/ofaf695.826

**Published:** 2026-01-11

**Authors:** Christian Rauschert, Abda Mahmood, Andreas Freitag, Daniel Heinze, Liu Zhang, Nishant Mehra, Pavo Marijic, Alexander Heiseke, Pavitra Dewda

**Affiliations:** GlaxoSmithKline GmbH & Co. KG, Munich, Bayern, Germany; GlaxoSmithKline GmbH & Co. KG, Munich, Bayern, Germany; Cytel, London, England, United Kingdom; GSK, Munich, Bayern, Germany; Cytel, London, England, United Kingdom; Cytel, London, England, United Kingdom; GSK, Munich, Bayern, Germany; GSK, Munich, Bayern, Germany; GSK, Munich, Bayern, Germany

## Abstract

**Background:**

Pertussis remains a significant public health concern, particularly for newborns and infants who are at a high risk of severe disease and mortality. Many countries have updated their national immunization schedules to include maternal booster vaccination to confer passive immunity to infants. This literature review aimed to evaluate the impact of maternal tetanus, diphtheria, and pertussis (Tdap) booster vaccination on the protection of newborns and infants.

**Table 1.**
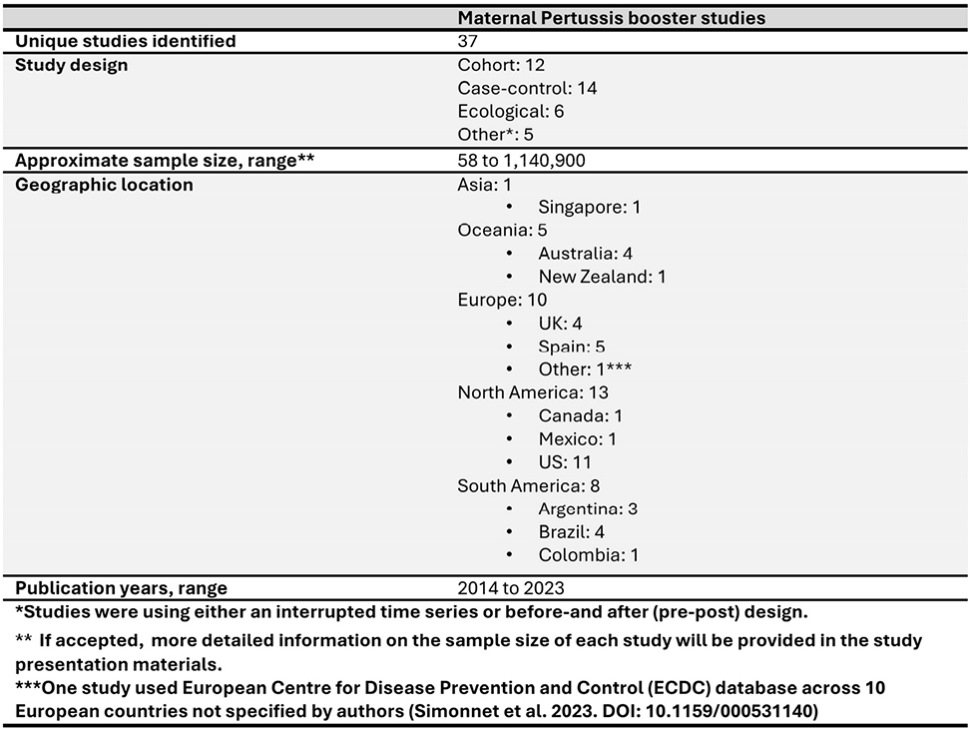
Characteristics of Studies on Maternal Pertussis Booster Vaccination Identified through Systematic Literature Review

**Table 2.**
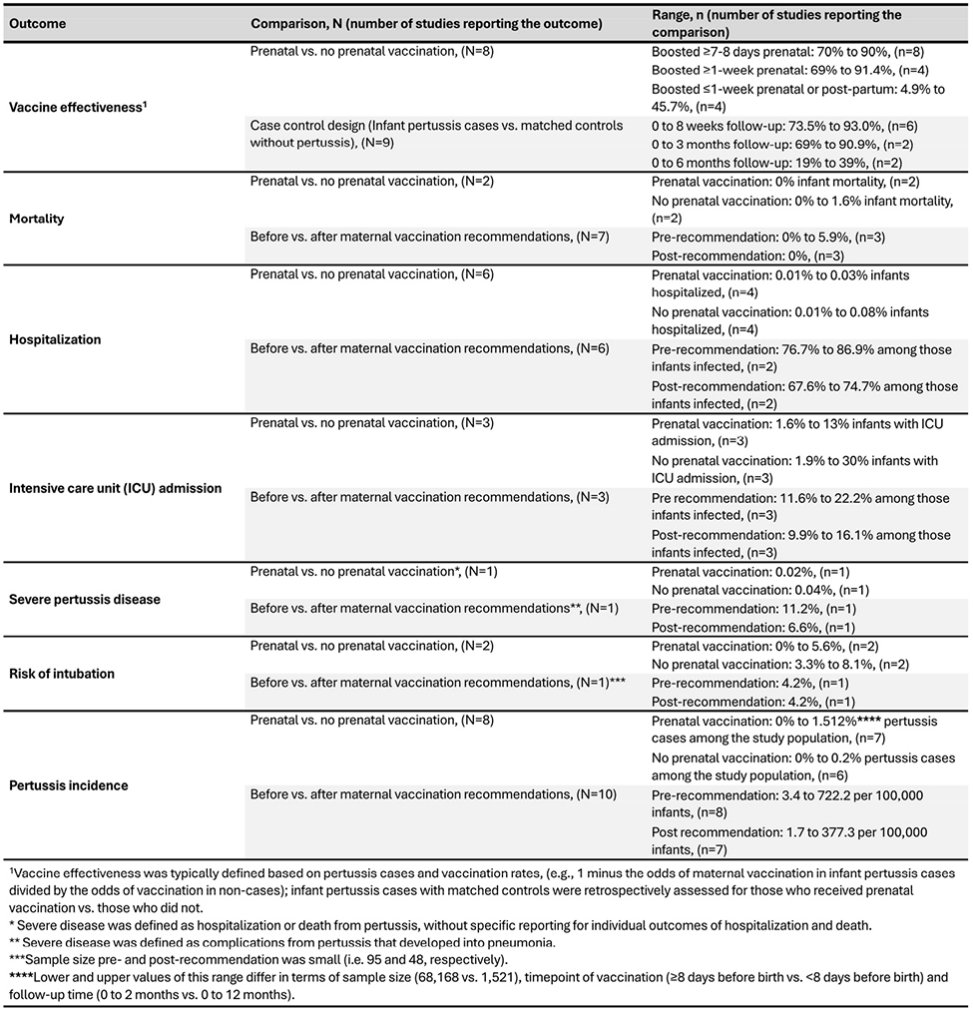
Summary of key outcomes from studies included in the Systematic Literature Review on pertussis vaccination during pregnancy stratified by comparator group

**Methods:**

A systematic literature search was conducted in MEDLINE, Embase, and Cochrane databases from inception to January 24, 2024. Studies comparing the effects of prenatal pertussis vaccination to no prenatal or postpartum vaccinations, were included. Data were extracted on various outcomes, including vaccine effectiveness (VE), risk of severe disease, risk of intubation, mortality, hospitalization rates, incidence rates and intensive care unit (ICU) admission. Articles in English and German were included.

**Results:**

A total of 37 studies (38 publications) conducted in various geographic locations were included (Table 1). VE of prenatal Tdap booster was highest when vaccination occurred ≥1 week before birth (69.0% to 91.4%; n=4 studies) versus ≤1 week before birth or postpartum (4.9% to 45.7%, n=4 studies) (Table 2). Two studies comparing prenatal with no prenatal vaccination reported 0% and 1.6% infant mortality in the prenatal and no-prenatal Tdap groups, respectively. Infant mortality and pertussis incidence rates decreased among infants after the introduction of prenatal immunization (Table 2). Prenatal Tdap vaccination reduced the risk of severe disease and intubation in infants aged 0 to 2 months and was associated with lower rates of hospitalization and ICU admission compared to no prenatal vaccination (Table 2).

**Conclusion:**

Our findings highlight the potential of Tdap boosters during pregnancy to enhance maternal immunization strategies, leading to significant decreases in pertussis incidence, severity, and mortality rates in infants.

**Disclosures:**

Christian Rauschert, PhD, GlaxoSmithKline GmbH & Co. KG: Employee Abda Mahmood, PhD, GSK: Employee Andreas Freitag, Medical Doctor, Cytel: Employed by Cytel at the time of the research|Roche Products Ltd: Employed by Roche Products Ltd - No financial interest in the work conducted/presented Daniel Heinze, PhD, GlaxoSmithKline: Employee|GlaxoSmithKline: Stocks/Bonds (Public Company)|Sanofi-Aventis: Stocks/Bonds (Public Company) Liu Zhang, n/a, Cytel: Advisor/Consultant Nishant Mehra, n/a, Cytel / GSK: Employed by Cytel - Cytel received research funding form GSK Pavo Marijic, PhD, GSK: employee|GSK: Stocks/Bonds (Public Company) Alexander Heiseke, PhD, GlaxoSmithKline GmbH & Co. KG: Employee|GlaxoSmithKline GmbH & Co. KG: Stocks/Bonds (Private Company) Pavitra Dewda, MBBS, MD, GSK: Employee|GSK: Stocks/Bonds (Private Company)

